# Fungal pretreatment of raw digested piggery wastewater enhancing the survival of algae as biofuel feedstock

**DOI:** 10.1186/s40643-016-0136-2

**Published:** 2017-01-12

**Authors:** Junying Liu, Wen Qiu, Yunpu Wang

**Affiliations:** 1 The Engineering Research Center for Biomass Conversion, Ministry of Education, Nanchang University, Nanchang, 330047 China; 2 State Key Laboratory of Rice Biology, Institute of Biotechnology, Zhejiang University, Hangzhou, 310058 China

**Keywords:** Fungi, Bacterial communities, NH_4_^+^, Turbidity, Algae, Digested piggery wastewater

## Abstract

**Background:**

Understanding about the impact of white rot fungi on indigenous bacterial communities, NH_4_^+^ and turbidity in digested piggery wastewater, will allow the optimization of wastewater treatment methods and its use as a feasible medium for algal growth. Here, the white rot fungi were inoculated into undiluted and unsterilized digested piggery wastewater under different temperatures and pH regimes in order to lower the pretreatment cost. Diversity and abundance of the bacterial communities in the pretreated wastewater were assessed by PCR-denaturing gradient gel electrophoresis coupled with 16S rDNA sequencing.

**Results:**

The research showed a significant reduction on the microbial diversity with the presence of white rot fungi which occur at pH 6. The distribution and presence of bacteria taxa were strongly correlated with NH_4_^+^ concentration, pH, and the presence of white rot fungi. Variance partition analysis also showed that the effect on the chlorophyll content of algae in fungi-filtered wastewater was as the following hierarchy: bacterial diversity > NH_4_^+^ > turbidity. Therefore, the algae in treated wastewater with less abundance of bacteria proliferated more successfully, indicating that bacterial community not only played an important role in algal growth but also imposed a strong top-down control on the algal population. The algae grown in wastewater treated with fungi reached the highest specific growth rate (0.033 day^−1^), whereas the controls displayed the negative specific growth rate. The fatty acid composition varied markedly in C16:0 and C18:0 between these treatments, with a higher content of C16:0.

**Conclusions:**

This study firstly showed that *Chlorella* can grow as cost-effective biofuel feedstocks in undiluted and unsterilized digested wastewater with high ammonium concentration and dark brown color because the bacterial abundance of digested piggery wastewater could be reduced greatly by the white rot fungi.

**Electronic supplementary material:**

The online version of this article (doi:10.1186/s40643-016-0136-2) contains supplementary material, which is available to authorized users.

## Background

One approach for biofuel production is based on the utilization of algal biomass harvested from the application of an algae-based method to treat agricultural wastewater or manure effluent (Craggs et al. 2013). Thus, an efficient and economically viable alternative pretreatment should be employed to treat digested effluent before being used for large-scale algal cultivation (Chen et al. 2011). The toxicity of many pollutants lessens the remediation efficiency of most algae but white rot fungi can withstand most of these toxic levels. Current physical and/or chemical methodologies are effective for decolorization of wastewater, but these methods are unsuitable for large-scale use, owing to their high cost, low efficiency, and poor adaptability to a wide range of pollutants (Waghmode et al. 2011). Moreover, high energy requirements and the secondary pollution problems in the form of sludge inhibit their wide application (Waghmode et al. 2011). Compared to chemical and/or physical methods, biological processes have received much more attention due to their cost-effectiveness, lower sludge residue, and eco-friendliness (Waghmode et al. 2012). The effectiveness of microbial decolorization depends on the adaptability and activity of the chosen microorganisms and the characteristics of the nutrient itself. Therefore, many factors such as strain selection, microbial ecology, and any environmental constraints must be taken into account to improve the biodegradation of digested piggery wastewater (Tyagi et al. 2011). Treatments with white rot fungi offer the possibility to expand the substrate range of existing treatments via biodegradation that cannot be removed by chemical reagents. Furthermore, several studies have also demonstrated that the use of white rot fungi is safer (e.g., non-hazardous) and eco-friendly compared to the conventional use of chemicals (Fulekar et al. 2013). Fungi perform more efficiently in the decolorization of wastewater compared to bacteria which require preconditioning to the particular pollutant (Garg and Tripathi 2011; Pakshirajan and Radhika 2013). *Phanerochaete chrysosporium* (PC) is the most investigated species and has been shown to be very promising for treating effluents from pulp and paper, coal conversion, textile, and olive oil industries (Taccari et al. 2009). It has now become a model species in studying pollutant bioremediation, especially in the decolorization of different dyes (Moredo et al. 2003). The pH has a significant impact on the decolorization potential of white rot fungi (Kiran et al. 2012; Pratt et al. 2012). However, due to the significant modifications on the microbial community with the presence of white rot fungi, it is difficult to distinguish the effects attributable to bacterial abundance or those inherent to the sample on algal growth in digested swine wastewater.

Another important composition of the medium is the bacterial communities which may act as algal diseases to some extent, especially when considering alternative water sources like wastewater for algal cultivation. Indigenous bacterial communities naturally mediate many ecological processes and play fundamental roles in the degradation of pollutants present in a local environment, i.e., swine wastewater (Zhang et al. 2013). Knowledge on how bacterial communities respond to different environmental conditions can provide deeper understanding of the parameters useful to control waste degradation and removal using microorganisms. Substantial research indicates that different physico-chemical factors affect bacterial communities in different extents. According to previous researches, the white rot fungi have shown selective effects on indigenous bacterial communities by competing for degradable substrates or changing the sample properties (e.g., pH, temperature, and carbon sources). The usage of bacteria in wastewater for algal cultivation has not been closely considered because, on one hand, bacteria usually occur in low density due to the high dilution and, on the other hand, they are unlikely to have no significant effects on algal growth due to sterilization in lab scale (Bartley et al. 2014). So far, there are no studies that have closely looked into the effects of bacterial community on algae growth in digested and non-sterilized fungal-pretreated swine wastewater. Compared with the cultivation-based techniques, PCR-DGGE and 16S rDNA sequencing offer comprehensive and high-resolution analytical advantageous means to investigate the bacterial communities (Dong and Reddy 2010).

This study then aimed to determine the optimum conditions (e.g., NH_4_^+^, turbidity, and bacterial abundance) for wastewater to be useful as an algal growth medium pre-treated with white rot fungi. Many researches have also demonstrated that *Chlorella* species are an ideal candidate for biofuel production owing to its high adaptability to wastewater and ability to inhibit bacterial growth by chemical defenses. *Chlorella vulgaris* is considered as one of the promising strains for lipid production due to its high growth rate and relatively high lipid content (Shu et al. 2012). Thus, in this study, the ability of *Chlorella* species to survive in the optimized wastewater with fungal removal was also tested.

## Methods

### Preparation of fungal inoculant and medium

The PC strain ATCC 24725 was provided by the China Center for Type Culture Collection (CCTCC; Wuhan, China). Test strain of PC was transferred to sterile PDA slants and stored at 4 °C before use. To produce enough fungus, the inoculant was grown in 200-mL sterile broth in 500-mL Erlenmeyer flasks with a 0.5 cm^2^ agar plug and then incubated at 30 °C in a rotary shaker at 135 rpm for 8 days. After which, 5 mL of the cultures containing fungal pellets of different sizes was filtered through a 0.45-µm nylon filter. The filtrates containing the fungi pellets were homogenized using sterile glass beads (8 mm) before use in effluent treatment. The moisture weight of inoculant mycelium pellets was 0.5 g mL^−1^. Before being applied for the subsequent studies, the wastewater was stored at low temperature (4 °C) to prevent natural degradation. Characteristics of this wastewater are shown in Table 1.

**Table 1:** Characterization of initial effluents from piggery anaerobic digestion

Parameter	Pre-treated value (mean ± SD)	After treatment value^a^
pH	8.6 ± 0.2	7.2 ± 0.5
Color (Abs. 400 nm)	1.1 ± 0.08	0.6 ± 0.07
TSS (mg L^−1^)	1133.3 ± 68.1	254.2 ± 31.7
COD (mg L^−1^)	625.8 ± 16.9	262.5 ± 12.6
TN (mg L^−1^)	330.6 ± 14.3	255.9 ± 10.9
TP (mg L^−1^)	50.3 ± 6.7	29.6 ± 8.5
NH_4_^+^ (mg L^−1^)	287.1 ± 25.2	226.6 ± 19.4

^a^The average data of 6 pretreated wastewater by fungi

### Experimental set-up and sample collection

Preliminary experiments showed that pH, temperature, and the amount of inoculum are the most significant variables affecting efficiency of effluent pre-treatment (Ye et al. 2014). The relative influence of the variables in the experiment is shown in Table 2. A total of 8 experimental conditions were tested. A final volume of 250 mL piggery wastewater was used and inoculated with different amounts of fungi in 500-mL flasks. Cultures were grown for 9 days under 24-h light (100 μmol photon m^−2^ s^−1^) provided by cool, white fluorescent tubes, and two different temperatures (i.e., 20 and 25 °C). Cultures were maintained under static conditions without aeration. The pH condition of the media was modified or maintained by adding either HCl or NaOH. All experiments were carried out in triplicate. Finally, the wastewater was used to grow algal cells after filtering by using a screen filter (0.66 mm) to remove fungi followed with fungus treatments at different temperatures (i.e., 20 and 25 °C) according to the experimental design (Table 2).

**Table 2:** Environmental parameters used in digested piggery wastewater and Shannon-Wiener

Code	Fungi	pH	Temperature	Shannon-Wiener
PC1	*Phanerochaete chrysosporium*	4	20	2.8161
PC2	*Phanerochaete chrysosporium*	4	25	2.8335
PC3	*Phanerochaete chrysosporium*	6	20	2.2597
PC4	*Phanerochaete chrysosporium*	6	25	2.5019
PC5	*Phanerochaete chrysosporium*	8	20	2.6528
PC6	*Phanerochaete chrysosporium*	8	25	2.4781
CK1	No fungal inoculum	8.6	20	2.8870
CK2	No fungal inoculum	8.6	25	2.6138

The *C. vulgaris* (FACHB 25) used in this study was purchased from the Freshwater Algal Culture Collection of the Institute of Hydrobiology, the Chinese Academy of Sciences (Wuhan, China). The algal inoculum was grown in 500-mL flasks containing 200 mL BG11 medium. The cultures were incubated in chambers illuminated at the light density of 100 µmol m^−2^ s^−1^ using cool white fluorescent lamps under a 12-h/12-h light–dark cycle at temperature regimes described in the aforementioned sections. The pH of the medium was adjusted to 7.2.

### Characterizing the samples

Physico-chemical characteristics (NH_4_^+^, total nitrogen (TN), phosphorous (TP), COD, and turbidity) of the samples throughout the study were determined following protocols described in the Standard Methods for the Examination of Water and Wastewater (APHA 2005). The kinetics coefficients for TN and TP have been calculated in this study according to the previous studies (Liu 2012; Wang et al. 2014). Algal biomass was measured by means of dry weight and chlorophyll content. Samples (10 mL) of cultures were centrifuged at 6000*g* at 4 °C for 5 min. Pellets were washed twice with distilled water and centrifuged again to remove impurities. Pellets were freeze dried for two days to determine dry weight. Chlorophyll content was also generally used as algal biomass and differentiates contribution from heterotrophs such as bacteria and fungi. The chlorophyll content was determined using modified spectrophotometric methods (Wellbum 1994). In brief, 2 mL of sample was collected every day from the treatments, and the pigment was extracted by acetone and quantified using a spectrophotometer (Thermo UV–Vis). The specific growth rate was determined as described in the previous study based on the change of chlorophyll content (Liu 2015). Lipid and protein contents of algae were determined as described in the previous study (Liu 2015; Volker et al. 1979). The carbohydrate was analyzed according to the method (DuBois et al. 1956). The fatty acid compositions were analyzed as described in the previous study (Liu 2015).

### The kinetics coefficient for total nitrogen (TN) and phosphorous (TP)

The relationship between the specific growth rate of *C. vulgaris* in shake flasks and nutrient concentration was investigated by estimating the parameters of the Monod equation (Doran 1995), a homologue of the Michaelis–Menten expression (Eq. 1):

1 $$\mu = \frac{{\mu_{\rm{max} } S}}{{K_{S} + S}},$$

where *S* is the concentration of the growth-limiting substrate; $\mu_{\rm{max} }$ is maximum specific growth rate (day^−1^); and $K_{S}$ is a substrate constant with the same dimensions as substrate concentration that is the substrate concentration at which the growth rate is half its maximum value.

The equation was transformed as follows into a suitable form for a straight line plot

2 $$\frac{1}{\mu } = \frac{{K_{S} }}{{\mu_{\rm{max} } }} \times \frac{1}{S} + \frac{1}{{\mu_{\rm{max} } }}.$$

According to this regression Eq. (2), the maximum specific growth rate (*µ*_max_) for the nitrate as growth-limiting substrate and substrate constant (*K*_*S*_) were calculated (Liu 2012).

### Genomic DNA extraction and PCR-DGGE

Fifty milliliters of wastewater was concentrated to 2-mL samples for genomic extraction by centrifugation at 8000 rpm. The genomic DNA of the microbial community from 2-mL samples was extracted using DNA Kits (Qiagen, Germany). Extracted DNA was quantified by a 200 microplate reader (Tecan, Switzerland). The V3 region of bacterial 16S rDNA was amplified using the universal primers 338F and 534R, with a GC clamp of 39 bases added to the 5-terminus. PCR amplification was performed in 50 µL reaction mixtures according to the methods described in previous study (Zhao et al. 2012). Denaturant Gradient Gel Electrophoresis (DGGE) was carried out to further analyze diversity of the amplified fragments of the 16S rDNA bacterial gene, and PCR products were placed under denaturing conditions as described in previous study (Zhao et al. 2012). The gels were run with 40 µL and were silver stained after (DuBois et al. 1956; Volker et al. 1979). Targeted bands of the 16S rDNA corresponding to possible different species were further isolated and purified using purification kits (Watson Biotechnologies, China) and cloned into *E. coli* DH5α. Three clones were randomly selected from each band, followed by PCR amplification of the cloned inserts (Zhao et al. 2012). Sequencing was performed as described in previous research (Xu et al. 2011).

### Data analysis

Generated 16S rDNA gene sequences were compared against GenBank database to obtain closely related sequences by BLASTn search. All hits with >97% similarity were downloaded and aligned with the unknown sequences for phylogenetic analysis (Matsunaga et al. 2005). Phylogenetic trees were constructed in MEGA 4.0. DGGE bands were analyzed to study the relatedness of the microbial communities with similarity coefficients. Two bands were considered to be related if they migrated the same distance on the DGGE gel (Patil et al. 2010; Zhang et al. 2008). Relative band intensities or peaks on DGGE community profiles were also determined. Each band was considered to be an operational taxonomic unit or species, and the band densities as proxy for abundance, which were then used to calculate the Shannon–Weaver diversity (H′) and equitability indices (J) (Shannon 2001). The noise levels and minimum peak thresholds of the software were set to optimum values in order to reduce background noise which only allowed the detection of genuine peaks (Patil et al. 2010).

Canonical correspondence analysis (CCA) was used to determine multivariate relationships between DGGE banding profiles and environmental factors. The analysis was performed using CANOCO 4.5, and the significance was measured by Monte Carlo test using 1000 permutations. Analysis of variance (ANOVA) was done in SPSS 19.0. The main and interaction effects of turbidity and NH_4_^+^ concentration were determined. Duncan tests were applied to assess statistical differences between treatments at 95% confidence level (p < 0.05).

## Results

### Changes in wastewater treated with fungi

**Fig. 1:**
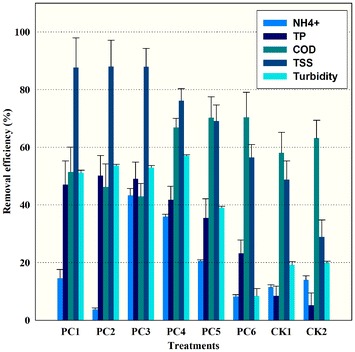
Nutrient removal efficiency (NH_4_^+^, TP, COD, TSS, and turbidity) of piggery wastewater by fungi at the end of experiment. PC1 (pH 4, 20 °C), PC2 (pH 4, 25 °C), PC3 (pH 6, 20 °C), PC4 (pH 6, 25 °C), PC5 (pH 8, 20 °C), PC6 (pH 4, 25 °C). CK1 (20 °C), and CK2 (25 °C) are controls. The initial physico-chemical characteristics are shown in Table 1

**C**hanges in the properties of the wastewater treated with fungi are shown in Fig. 1. Results suggested that pollutant removal (NH_4_^+^ and turbidity) was tightly dependent on the selection of environmental conditions. In this study, the removal of NH_4_^+^ seemed to vary with pH levels. Treatments with pH 4 resulted in the decrease of a more acidic condition of pH 3.56, while the treatment with pH 6 led to an increase of a more alkaline condition of around pH 8.5. The treatments with pH 8 caused a slight increase at the end of experiment to around pH 9. The percent removal of NH_4_^+^ increased with the rising of pH levels but the maximum removal effect (i.e., 43% by PC) occurred at conditions with an initial pH of 6. The maximum color reduction occurred in PC3 and PC4 with an initial pH of 6, while the minimum turbidity reduction (10%) occurred in PC2 with an initial pH of 4 at 25 °C, which was even less than the control (20%). As for turbidity reduction of total treatments, there were significant differences between fungus treatment and controls.

### Bacterial community diversity based on PCR-DGGE

PCR-DGGE was used to investigate the impact of the fungi on the structure of the indigenous bacterial community. Bands of the DGGE profile corresponding to different PCR-amplified 16S rDNA fragments were obtained from different species or strains (Table 3; Additional file 1: Figure S1). Each sample had distinct DGGE pattern, and bands from each lane showed big difference with each other. Bands were visible until approximately 50% denaturant. On one hand, the treatments with an initial pH of 6 inoculated with PC showed less bacterial diversity compared to the other treatments. On the other hand, the treatments with an initial pH of 4 with the presence of white rot fungi showed the most bacterial species diversity. It suggested that the bacterial communities were affected by pH condition. It was further supported by the data of lowest Shannon-Wiener index H′ value of 2.2597 for PC3, whereas the treatment with an initial pH 4 had the highest Shannon-Wiener index H′ value (2.8161 for PC1), which was close to the value of CK (2.8870).

**Table 3:** 16S rDNA gene sequence obtained from the pretreated wastewater by white rot fungi in this study

Clone No.	Best match database (Accession)	Similarity (%)	Environmental functions	References
S1	*Bacteroides* sp. (AB596884.1)	99	Break down macromolecules in anaerobic digesters	Auerbach et al. (2007)
S2	*Cloacibacterium normanense* (LN613116.1)	100	Anaerobic, rod-shaped bacterium to recycle fibers	Ntougias et al. (2015)
S3	*Petrimonas sulfuriphila* (NR_042987.1)	100	Exist in removal process of ammonium in the wastewater	Nakasaki et al. (2009)
S4	*Flavobacterium cucumis* (KF261012.1)	100	Degrade potentially toxic compounds	Spain et al. (2007)
S5	*Achromobacter* sp. (LK936601.1)	100	The ammonia and nitrite oxidizing bacteria	Honda and Osawa (2002)
S6	*Xenophilus* sp. (KC010298.1)	98	Nitrification treatment of methane fermentation digestion	Qiao et al. (2010)
S7	*Dechloromonas* sp.(EF632559.1)	97	Degrade phenol and benzene	Spain et al. (2007)
S8	*Stenotrophomonas* sp. (EF221774.1)	100	Efficiently degrade NO_3_–N in semi-anaerobic condition	Yu et al. (2009)
S9	*Uncultured Bacteroidetes bacterium* (KF630625.1)	99	Remove ammonium in the wastewater	Zhou et al. (2010)
S10	*Alcaligenes* sp. (FR745404.1)	99	Relatively high ammonium removal potential	Spain et al. (2007)
S11	*Bacillus* sp. (KC430992.1)	100	Remove ammonium in the wastewater	Zheng et al. (2011)
S12	*Castellaniella defragrans* (FJ982930.1)	100	Degrade phenol and benzene	Spain et al. (2007)
S13	*Denitrobacter* sp. (EF471227.1)	99	Degradation of wood and cycling of nitrogen and sulfur	Lu et al. (2003)
S14	*Magnetospirillum* sp. (*KM289194.1*)	100	Contribute to the global iron cycle	Matsunaga et al. (2005)

Environmental functions were derived from literature

Bacterial 16S rDNA sequences re-amplified from the dominant DGGE bands were further used for phylogenetic analysis (Table 3; Additional file 1: Figure S2). The phylogenetic tree showed that there were mainly four groups of bacterial species dominant in the wastewater. Most corresponded to bands S1, S3, S4, S6, S9, and S11, which were closely related to *Bacteroides* sp., *Petrimonas sulfuriphila*, *Flavobacterium cucumis*, *Xenophilus* sp., uncultured *Bacteroidetes*, *Bacillus* sp., with at least 99% similarity to the reference sequences. Bands S10 and S13 appeared only in treatments with pH 4, whose closest match (99%) was *Alcaligenes* sp. and *Denitrobacter* sp. (99%), respectively. The presence of these species corresponds well with the relatively high ammonium removal potential found in this treatment. However, since only abundant microbial populations can be detected by PCR-DGGE of 16S rDNA, it can be assumed that the *Achromobacter* sp. constitutes the majority of the ammonia and nitrite oxidizing bacteria in the studied treatments, implying the presence of these oxidizing species to the removal of pollutants (Table 3). Interestingly, only band S9 seemed to be different in DGGE profiles of microbial communities under the same initial pH level with fungal treatment. It was mostly similar to an uncultured *Bacteroidetes* with 99% similarity. In addition, bands S5 (ID, 99%) and S6 (ID, 99%) were the most distributed and appearing in all treatments. These species were characterized as ubiquitous predatory bacteria and thus were not expected to be specific to any treatment. This bacterium was found in all treatments, suggesting it possibly plays an important role in the removal of ammonium in the wastewater.

### Algal cells grown in different wastewater pretreated by fungi

**Fig. 2:**
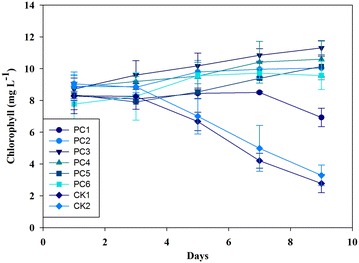
Chlorophyll concentration of algal cells grown in different pretreated wastewater by *Phanerochaete chrysosporium.* PC1 (pH 4, 20 °C), PC2 (pH 4, 25 °C), PC3 (pH 6, 20 °C), PC4 (pH 6, 25 °C), PC5 (pH 8, 20 °C), PC6 (pH 4, 25 °C). CK1 (20 °C), and CK2 (25 °C) are controls

**Fig. 3:**
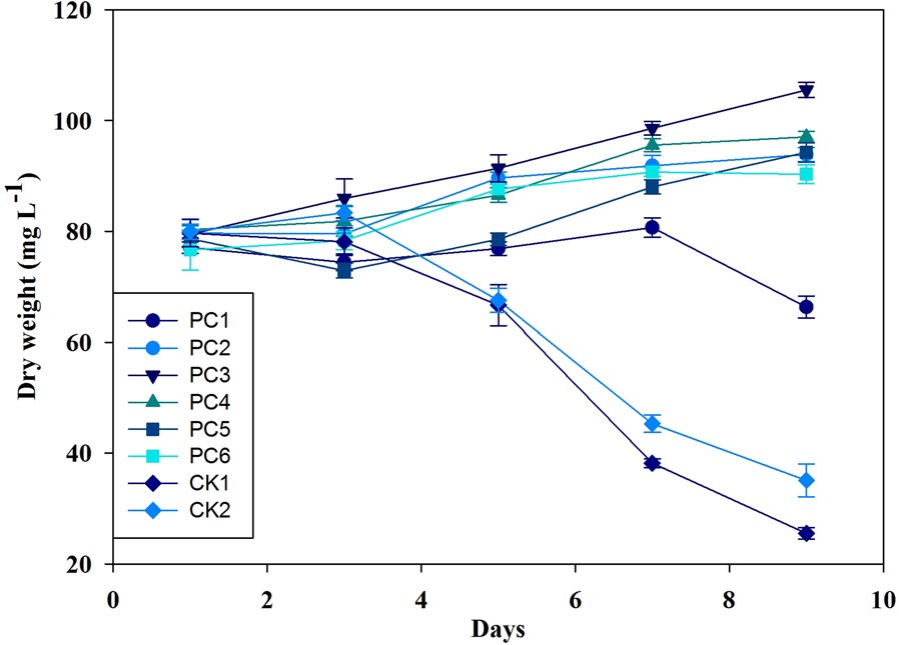
Dry weight of algal cells grown in different pretreated wastewater by *Phanerochaete chrysosporium.* PC1 (pH 4, 20 °C), PC2 (pH 4, 25 °C), PC3 (pH 6, 20 °C), PC4 (pH 6, 25 °C), PC5 (pH 8, 20 °C), PC6 (pH 4, 25 °C). CK1 (20 °C), and CK2 (25 °C) are controls

**Fig. 4:**
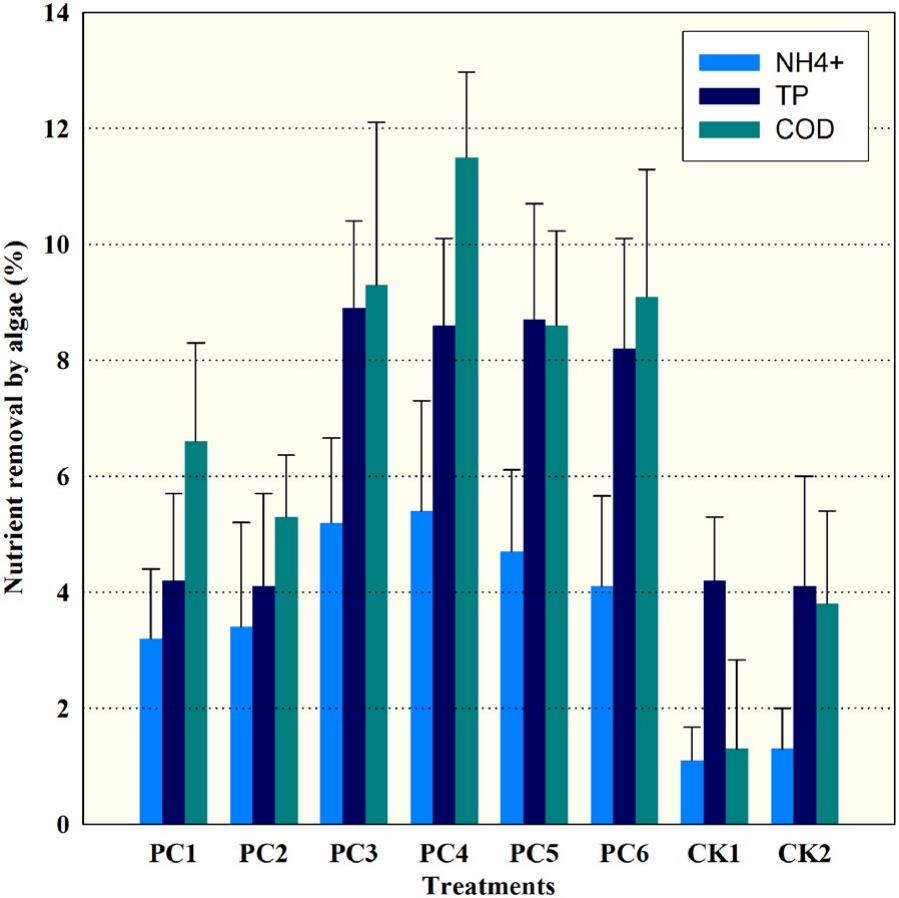
The removal of nutrients (NH_4_^+^, TP and COD) by algae after 9 days. PC1 (pH 4, 20 °C), PC2 (pH 4, 25 °C), PC3 (pH 6, 20 °C), PC4 (pH 6, 25 °C), PC5 (pH 8, 20 °C), PC6 (pH 4, 25 °C). CK1 (20 °C), and CK2 (25 °C) are controls

**Fig. 5:**
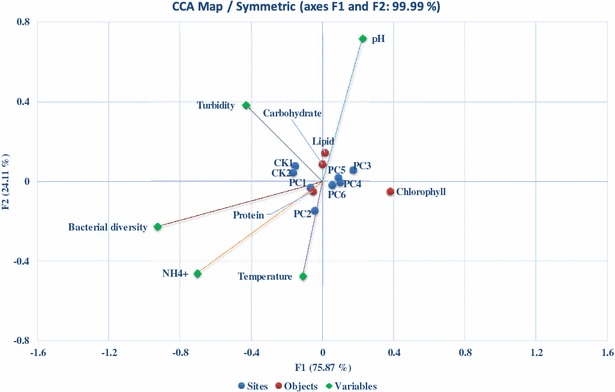
Algae-environment biplot from canonical correspondence analysis (CCA) summarizing differences in chlorophyll, protein, carbohydrate, and lipid of different treatments (PC1–PC6, CK1, CK2) and environmental variables (i.e., pH, temperature, NH_4_^+^, turbidity, and bacterial diversity). PC1 (pH 4, 20 °C), PC2 (pH 4, 25 °C), PC3 (pH 6, 20 °C), PC4 (pH 6, 25 °C), PC5 (pH 8, 20 °C), PC6 (pH 4, 25 °C). CK1 (20 °C), and CK2 (25 °C) are controls

The raw digested piggery wastewater after treatment by fungi was used to cultivate *C. vulgaris* (Figs. 2, 3). As expected, in the first few days, cells (indexed by chlorophyll content and dry weight) in pretreated wastewater showed a little growth compared to those in raw untreated wastewater. On the first three days, all treatments reached the stationary phase, but the CK in the raw untreated wastewater showed declined trend. In contrast, cells grown in PC3 of the treated wastewater reached a relative high biomass. A significant difference was observed among all treatments at the end of the study. All of algal cells in the treated wastewater showed a slight increase in chlorophyll concentration until day 7. PC1 and PC2 showed a slight decline in biomass (18%), other treatments in pretreated wastewater continued to grow and reached the maximum biomass potential at day 9, whereas CKs had the most dramatically declined in biomass (nearly 70%). Therefore, the PC3 reached the highest specific growth rate (0.033 day^−1^), whereas the CKs with the negative specific growth rate (−0.14 day^−1^). The nutrient removal efficiency was higher by algae in PC4 and PC3 than the other treatments (Fig. 4). The maximum specific growth rate (*µ*_max_) for the nitrate as growth-limiting substrate (TN) was 0.390 day^−1^ and the substrate constant (*K*_*S*_) was calculated as 14.23 mg L^−1^, while the value was 0.309 day^−1^ and 4.15 mg L^−1^, respectively, for TP. CCA ordination of chlorophyll and other environmental parameters also revealed that NH_4_^+^, turbidity, and bacterial community were strongly correlated with the first CCA axis (*r* = −0.081, −0.050 and −0.107, respectively), while temperature was the most correlated parameter with the second axis (Fig. 5). These two axes alone already explained 99.99% of total variance. Furthermore, CCA showed that all treatments were centered in map, indicating that there was other parameter which can distinguish these treatments (such as carbon sources).

### Effects of environmental parameters on the composition of the bacterial community

**Fig. 6:**
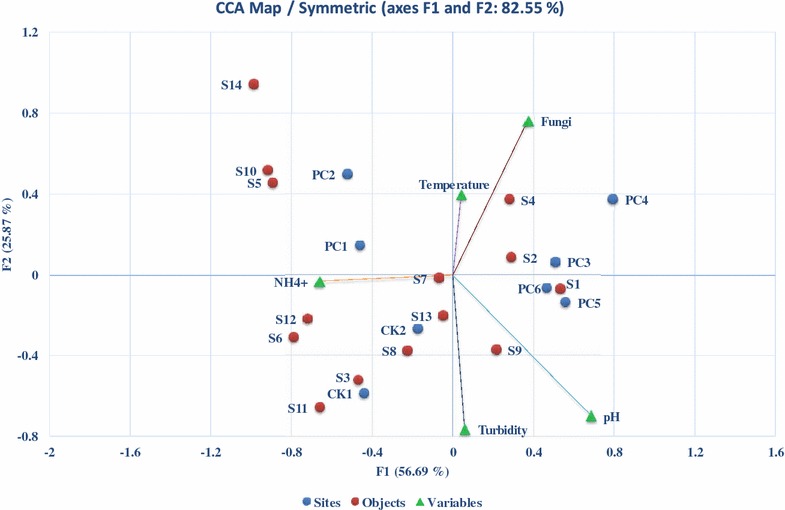
Species-environment biplot from canonical correspondence analysis (CCA) summarizing differences in bacterium species (S1–S14) of different treatments (PC1–PC6, CK1 CK2) along with environmental variables (i.e., pH, temperature, NH_4_^+^, turbidity, and fungal pretreatment. PC1 (pH 4, 20 °C), PC2 (pH 4, 25 °C), PC3 (pH 6, 20 °C), PC4 (pH 6, 25 °C), PC5 (pH 8, 20 °C), PC6 (pH 4, 25 °C). CK1 (20 °C), and CK2 (25 °C) are controls

Canonical analysis revealed that the first two factors (principal components) explained 82.55% (56.69% for F1 and 25.87% for F2) of the variability in species data (Fig. 6). The CCA ordination of the species and environmental variables demonstrated that NH_4_^+^, fungi, and the pH of the media were correlated with the first CCA axis (*r* = −0.328, 0.183 and 0.339, respectively). Whereas, pH was the most correlated parameter with the second axis (*r* = −0.255) and temperature was weakly correlated with the second axis (*r* = 0.133). The distribution of species was mainly related to NH_4_^+^, pH, turbidity, and the presence of fungi but most of the variation (39%) in the first axis was contributed by NH_4_^+^ in this experiment. However, the total explanatory effect of the environmental parameters was not significant as confirmed by the Monte Carlo permutation test (*p* > 0.05).

### Fatty acid composition of algal lipid

**Fig. 7:**
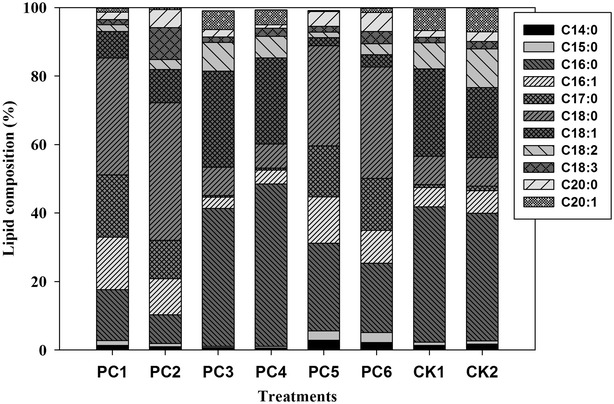
The fatty acid composition of *Chlorella vulgaris* after 9-day cultivation. PC1 (pH 4, 20 °C), PC2 (pH 4, 25 °C), PC3 (pH 6, 20 °C), PC4 (pH 6, 25 °C), PC5 (pH 8, 20 °C), PC6 (pH 4, 25 °C). CK1 (20 °C), and CK2 (25 °C) are controls

The lipids extracted after 9 days of cultivation from *C. vulgaris* was converted to fatty acid methyl ester (FAME), and their compositions are summarized in Fig. 7. We found that the major constituents composed of palmitic acid (C16:0), stearic acid (C18:0), oleic acid (C18:1), linoleic acid (C18:2), and linolenic acid (C18:3) regardless the growth conditions, such that the sum of these four fatty acid accounted for approximately 90% of the total fatty acids in the cells. However, the amount of saturated (C16:0 and C18:0), mono-unsaturated (C16:1 and C18:1), and poly-unsaturated (C18:2 and C18:3) fatty acids in the microalgae grown with different wastewater accounted for 8.32–47.45%, 3.25–28.12%, and 1.58–11.28% of the total fatty acid, respectively. The fatty acid composition varied markedly in C16:0 and C18:0 between these treatments, with a higher content of C16:0. The highest concentration of saturated fatty acid (47.45% of C16:0) was observed in wastewater treated by fungi at pH 6 and 25 °C. The lipid productivities of the algae were calculated in this study (PC1: 5.29, PC2: 21.57, PC3: 36.92, PC4: 24.32, PC5: 23.37, PC6: 20.80, CK1: 24.19, CK2: 28.88 mg L^−1^ day^−1^, respectively).

## Discussion

The optimization on more economic and eco-friendly ways for treating and recycling swine wastewater has attracted growing interest resulting from the potential utilization of raw wastewater for algal biofuel production (Table 4). The energy balance and economic viability of biodiesel production from algae improved with the application of wastewater, which results in 71% cost reduction of producing per ton of algal biomass (Olguín 2012). Our previous studies showed that high ammonium concentration and bacterial communities may inhibit algal growth. The pretreatment of the effluent becomes a challenge because of the requirement of non-toxic and eco-friendly methods compared to chemical reagents. Specially, the bacterial community is a critical issue needed to be addressed for pretreatment methods especially in digested effluents since the abundance of bacteria can control their host populations (Gachon et al. 2010). *P. chrysosporium* has already been used in treating digested effluents and capable of affecting the physical and biochemical properties of the wastewater (Liu et al. 2015). Results indicated a significant decrease on the microbial diversity with the presence of white rot fungi at pH 6. Therefore, to determine the factors significantly affecting the indigenous bacterial species is an important step to have a good understanding on changes in physico-chemical conditions during wastewater treatment. Direct multivariate statistics (e.g., CCA) provided powerful means to determine different parameters that affect the indigenous bacterial communities within different treatments. The results also indicated that NH_4_^+^, pH, and the presence of fungi as well as the interactions led to the most influence on the bacterial community when the fungi were introduced. It was inferred that the mechanism underlying this phenomenon may be because the fungus could influence the bacterial community structure indirectly through changing pH, reducing ammonium concentration, and competing for substrate and space. Moreover, changes in C/N ratio of wastewater also changed other physico-chemical parameters (e.g., pH, dissolved organic carbon, and total suspended solid), which in turn also affected the bacterial community, for instance, *P. chrysosporium*. Phylogenetic analyses revealed that the majority of the taxonomic groups were found to play a role in the nitrogen cycle. Most of the detected sequences were found to be related to *Bacteroides* genus, which had already been reported to be abundant in wastewater treatment plants before (Auerbach et al. 2007). The secondary taxonomic group is highly diverse and is well-known to comprise communities in anaerobic digesters where they break down macromolecules of feeding fermentation systems (Nakasaki et al. 2009).

**Table 4:** Comparison of nutrient removal and biomass and lipid production in microalgae grown in various wastewater conditions

Microalgal species	Wastewater type	Nutrient removal	Biomass production	Lipid content (%)	References
*Chlorella vulgaris*	Tertiary municipal wastewater	100%	0.29 g L^−1^	30	Ji et al. (2013b)
*Chlorella vulgaris*	Piggery wastewater effluent	68% N, 42% P,	1.1 g L^−1^	–	Ji et al. (2012)
*Chlorella vulgaris*	Treated domestic sewage	73.77% N, 100% P	–	7.6	Sydney et al. (2011)
*Chlorella sorokiniana*	Palm oil mill effluent	–	8.0 mg L^−1^ day^−1^	28.27	Putri et al. (2011)
*Scenedesmus obliquus*	Piggery wastewater effluent	26 mg L^−1^N, 1.9 mg L^−1^ P	0.18 g L^−1^	27	Ji et al. (2013a)
*Scenedesmus obliquus*	Tertiary municipal wastewater	100%	0.31 g L^−1^	27	Ji et al. (2013b)
*Scenedesmus obliquus*	Municipal wastewater	97% N, 98% P	0.41 wt L^−1^	22	Abou-Shanab et al. (2013)
*Scenedesmus* sp.	Electric factory wastewater	46% N, 100% P	4.5 g L^−1^	–	Su et al. (2011)
*Scenedesmus rubescens*	Synthetic wastewater	95.55% P	–	14.91	Aravantinou et al. (2013)
*Nannochloropsis salina*	Anaerobic digestion effluent	89% N, 82.8% P	155.3 mg L^−1^ day^−1^	24.9	Cai et al. (2013)
*Nannochloropsis Salina*	Diluted digester effluent	97% TN	204.12 mg L^−1^ day^−1^	32	Sheets (2013)
*Nannochloropsis* sp.	50% municipal wastewater	–	2.23 g L^−1^	59.9	Jiang et al. (2011)
*Micratinium reisseri*	Mine wastewater	97%	0.8 g L^−1^	17	Ji et al. (2014)
*Micractinium reisseri*	Municipal wastewater	–	0.26 wt. L^−1^	19	Abou-Shanab et al. (2013)
*Botryococcus braunii*	Treated domestic sewage	60.02% N, 51.15% P	1.88 g L^−1^	36.14	Sydney et al. (2011)
*Ourococcus multisporus*	Tertiary municipal wastewater	100%	0.31 g L^−1^	31	Ji et al. (2013b)
*Neochloris vigensis*	Synthetic wastewater	94.92% P	–	19.29	Aravantinou et al. (2013)
*Chlorococcum spec.*	Synthetic wastewater	94.34% P	–	6.93	Aravantinou et al. (2013)

A good review on the research before 2011 about the algal biofuel production using wastewater resources has been made by Pittman et al. (2011)

In addition, results also showed that bacterial abundance was the most limiting parameter for algal growth in untreated wastewater as they directly influenced photosynthetic activities, followed by NH_4_^+^ levels and turbidity. Therefore, the algae in treated wastewater with less abundance of bacteria proliferated more successfully, indicating that bacterial community played an important role in algal growth, imposing a strong top-down control on the algal population. Moreover, a similar growth rate of algae from all fungi-treated wastewater indicated that both of algae and bacteria were limited by the availability of carbon (i.e., CO_2_) such as the carbon source in wastewater (e.g., humic acid), which has been consumed up or degraded by fungi. The raw digested piggery wastewater with high concentration of ammonium and dark brown color, which has been used to grow algae with a high dilution (up to 20 times of dilution rate) was greatly different from the general municipal wastewater (Pittman et al. 2011). The high dilution rate would consume huge amount of freshwater which increase the cultivation cost. Therefore, this study firstly showed that *Chlorella* can grow as biofuel feedstocks in undiluted and unsterilized digested wastewater originally with high ammonium concentration and dark brown color because the bacterial abundance of digested piggery wastewater could be reduced greatly by the white rot fungi. The result also showed that the efficiency of nutrient removal (e.g., NH_4_^+^, TP and COD) was relatively poor for all the experimental variations but the TP uptake was strongly correlated with the microalgal growth. Because the NH_4_^+^ stripping increased the TN uptake, the differentiation of TN removal between biological uptake and abiotic precipitation could be carried out according to the theoretical formula of microalgae as shown in previous study (Ji et al. 2012; Wang et al. 2014). Therefore, the result demonstrated again that the bacterial abundance was the most limiting parameter for algal growth in untreated wastewater. However, the fatty acid compositions were affected mostly by pH and temperature. The results showed that comparatively higher saturated fatty acid compositions (C16:0 and C18:0) were found at pH 6 and 25 °C, while the polyunsaturated fatty acids had a higher amount at 20 °C than that at 25 °C. The differences in saturated fatty acid contents and C-chain length would cause noticeable change in the biodiesel properties. Relatively higher content of polyunsaturated fatty acid in algal oil causes deterioration in the quality of biodiesel upon storage (Zhang et al. 2008).

## Conclusion

This study demonstrated that the introduction of fungal species induced changes in the indigenous microbial community of the swine wastewater through affecting its pH and nutrient concentrations. On one hand, canonical correspondence analysis showed that fungi inoculation provided direct evidence that the contribution of the variation in the bacterial community, whereas the wastewater property changes induced by different inoculated conditions also contributed to algal growth to some extent. Variance partitioning, on the other hand, revealed that the bacterial community played an important role in algal growth, which was supported by the observation that the algal cells could survive in undiluted digested piggery wastewater pretreated with fungi in comparison to cells in untreated wastewater which had a decline of 70% in biomass. The algae grown in wastewater treated with fungi reached the highest specific growth rate (0.033 day^−1^), whereas the CK showed a negative specific growth rate. The fatty acid composition varied markedly in C16:0 and C18:0 between these treatments, with a higher content of C16:0. Therefore, this was the first study showing that the bacterial diversity could be reduced greatly by the white rot fungi and then the algae can grow in undiluted and unsterilized digested wastewater.

## Additional file

**Additional file 1.** Phylogenetic tree associations of microbial populations and DGGE profiles of amplified 16S rDNA fragments.
