# Supplementary material for: Fungal pretreatment of raw digested piggery wastewater enhancing the survival of algae as biofuel feedstock
Source: Bioresour Bioprocess. 2017 Jan 12;4(1):6. doi: 10.1186/s40643-016-0136-2 (PMC5236085; doi:10.1186/s40643-016-0136-2)
Supplement: Supplementary file 1 — Additional file 1. Phylogenetic tree associations of microbial populations and DGGE profiles of amplified 16S rDNA fragments. [file 40643_2016_136_MOESM1_ESM.docx]

Figure S1. Phylogenetic tree associations of microbial populations in the pretreated wastewater by two fungi. Phylogenetic tree showing the relations of sequences obtained from excised bands from the wastewater. The tree was inferred with the neighbor-joining method using a 70% similarity cutoff filter and Olsen correction method by Mega3.1 software. Scale bar 1% estimated difference in nucleotide sequence position.


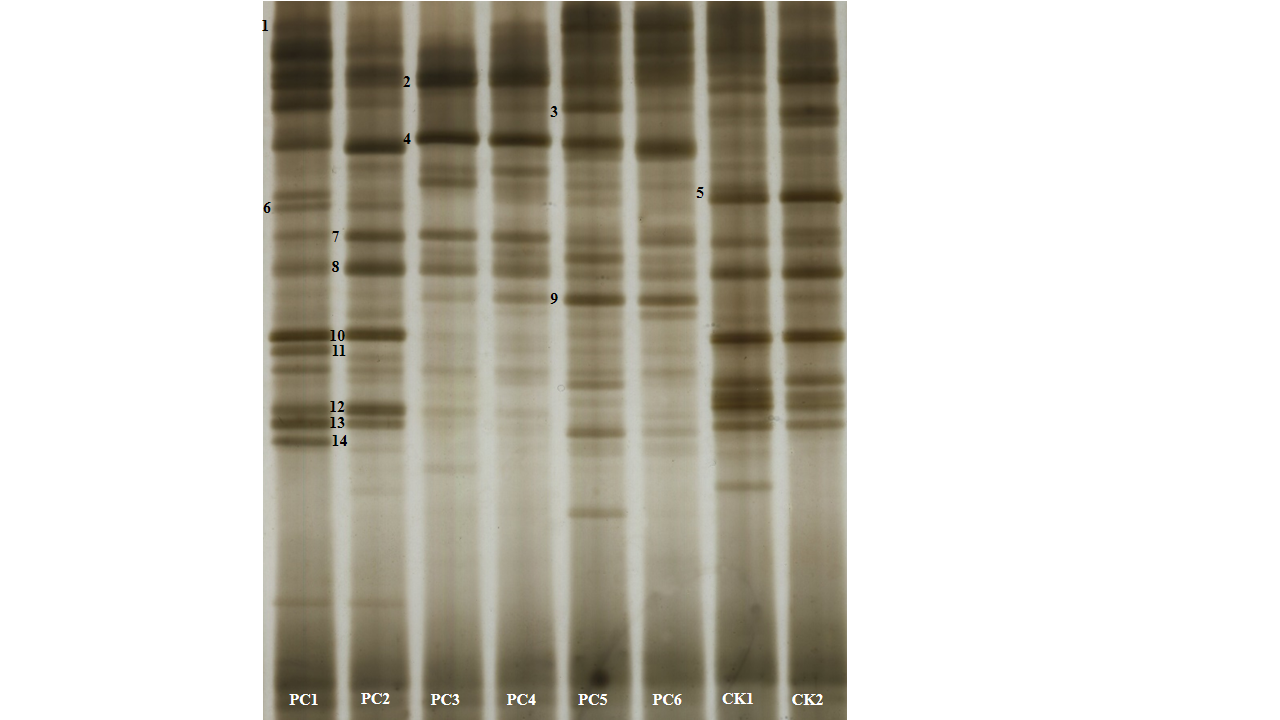


Figure S2 DGGE profiles of amplified 16S rDNA fragments of total bacterial population from wastewater pretreated by fungi. PC1 (pH4, 20℃), PC2 (pH4, 25℃), PC3 (Ph6, 20℃), PC4 (pH6, 25℃), PC5 (pH8℃, 20℃), PC6 (pH4, 25℃). CK1 (20℃) and CK2 (25℃) are controls. The numbers (1-14) on the picture represent clone No. (S1-S14).
